# Technostress of Chilean Teachers in the Context of the COVID-19 Pandemic and Teleworking

**DOI:** 10.3390/ijerph18105458

**Published:** 2021-05-20

**Authors:** Carla Estrada-Muñoz, Alejandro Vega-Muñoz, Dante Castillo, Sheyla Müller-Pérez, Joan Boada-Grau

**Affiliations:** 1 Departamento de Ergonomía, Universidad de Concepción, Concepción 4070386, Chile; carlaestrada@udec.cl; 2 Public Policy Observatory, Universidad Autónoma de Chile, Santiago 7500912, Chile; sheyla.muller@uautonoma.cl; 3 Centro de Estudios e Investigación Enzo Faletto, Universidad de Santiago de Chile, Santiago 9170022, Chile; dante.castillo@usach.cl; 4 Departamento de Psicología, Universidad Rovira i Virgili, 43007 Tarragona, Spain; joan.boada@urv.cat

**Keywords:** mental health, technostress, education, dark side, information overload, skepticism, fatigue, anxiety, inefficacy, confirmatory factor analysis

## Abstract

This article shows the levels of technostress in primary and secondary education teachers in Chile, in the context of educational telework that Chile has adopted in connection with the health crisis by COVID-19. The information has been collected with the use of the RED-TIC scale, previously used in this country, whose validity and reliability of the instrument has been treated, for this case, with confirmatory factorial analysis (CFA) with a national coverage sample of 3006 teachers. The results show that 11% of teachers reveal techno anxiety and 7.2%, techno fatigue. Combining both manifestations, we find that 6.8% of teachers are techno stressed. Finally, fatigue and anxiety factors are higher for female teachers.

## 1. Introduction

**Figure 1:**
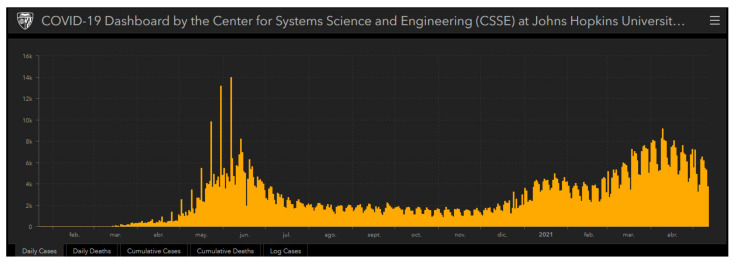
SARS-CoV-2 daily cases in Chile.

The objective of this research is to measure the stress levels associated with the use of information and communication technologies (ICTs) and identify the existence of differences according to gender, in primary and secondary education teachers in the context of the COVID-19 pandemic, in Chile, where the complete closure of schools was implemented as sanitary measure. The importance of this study is its contribution to knowledge on the subject, as a basis for the formulation of strategies and measures that allow the effective and sustainable integration of ICTs in the educational field, which last beyond COVID-19 pandemic since the available evidence is scarce. In the case of Chile, COVID-19 pandemic has been a long-lasting phenomenon given that as reported by the COVID-19 Dashboard by the Center for Systems Science and Engineering (CSSE) at Johns Hopkins University, the occurrence time between the first and second wave was 10 months [1] (See Figure 1).

The demands and resources of work in the educational field, where primary and secondary school teachers develop, have an important role in stress and exhaustion. Work in public middle schools, and especially in urban areas, are related to greater manifestations of stress and exhaustion on teachers, due to high labor demands and scarce resources, and stress with lower levels of education. The emotional exhaustion of teachers is associated with high turnover rates and lower quality teaching, which impacts student participation and performance. The promotion of the school’s organizational health, personal self-confidence, affiliation with colleagues, and having more resources academically, such as preventive interventions, are associated with less stress and exhaustion [2,3].

In a study developed by Alvites-Huamaní [4] on teaching stress and psychosocial factors in basic and higher education teachers in Latin America, North America, and Europe, a significant positive correlation was found between stress and psychosocial factors such as workplace conditions, workload, content and characteristics of duty, academic role and career development, social interaction, and organizational aspects. According to Mondal et al. [5] in their study on stress and job satisfaction in teachers, they found that they were partially satisfied and experienced stress levels from mild to moderate in their work, probably because of unfavorable working conditions, existing the need for more support and recognition from the institution.

According to research by Agai–Demjaha et al. [6], among the main stress-causing factors in teachers are the change in terms and conditions related to work without prior consultation, granting responsibilities, but without the authority to make decisions, the lack of resources to carry out the work, and limited access to training. The highest levels of stress in the face of changes in education are manifested in primary, older, and university-trained teachers. The highest stress levels in the face of a lack of authority to make decisions are manifested in primary school teachers, women, who work in their first job and with university education. High school teachers, who work in their first job and with university education, most often receive stress from lack of resources to do their job. As reported by Von der Embse et al. [7], the high stress level of teachers has an impact on students’ mental and behavioral health, and therefore on school outcomes, making it relevant to implement effective intervention for stress management, such as allocating resources for classroom management training or student behavior management.

The integration of ICTs into education in recent years has become relevant, involving a transformation in how work is organized, requiring teachers to possess the skills to use and incorporate ICTs as a teaching and learning tool, which has caused stress associated with their use, called technostress. Technostress was originally defined as a condition resulting from an individual or organizational inability to adapt in a healthy way to the use of new technologies, which is modulated according to age, previous technological experiences, workload, perception of control and work climate, and, consequently, it affects people’s performance, thus limiting their use of technology [8]. According to Tarafdar [9], it corresponds to the stress that people experience, due to the use of ICT, derived from the demands that these cause on the individual. Techno-anxiety is the best-known type of technostress, where the person experiences high levels of unpleasant physiological activation and feels tension and discomfort due to the present or future use of some type of ICT. The same anxiety leads to skeptical attitudes regarding the use of technologies, as well as negative thoughts about their own capacity and competence with ICT, and on the other hand, techno-fatigue is characterized by feelings of fatigue and mental and cognitive exhaustion due to the use technologies, also complemented by skeptical attitudes and beliefs of ineffectiveness with the use of ICT [10].

The stress associated with the use of ICT has been studied in different contexts [11], including at the level of university education [12,13]. Among the main reasons teachers experience techno-stresses are individual problems, technical problems, education-oriented problems, health problems, and time problems. There are differences between women teachers and man teachers; in women, technostress is mainly associated with technical problems involving the need for technical support, software, and connection problems; instead in men, first, it is related to individual problems, such as self-efficacy and attitude towards technology use, and the economic situation [14].

Teachers who teach using video conferencing technology would be under constant scrutiny, with labor demands that can trigger in techno-stresses [15]. Thus, workers’ acceptance of incorporating these new technologies into an organization to promote their effective use and well-being is relevant. Workers need to be involved in this transformation process, having opportunities where they are communicated and receive information on the implementation of technologies; receive training to improve their knowledge and skills in their use; and participate in specific training courses [16]. In a study on the use of social media at work, it was found that when workers see the personal advantage of using them, they tend to use them more, compared to when the employer requires them to use them. In this sense, it is essential to provide training to workers, as this contributes to improving their skills, having better regulatory mechanisms, fostering opportunities to establish meaningful connections with others, producing intrinsic motivation, and having a good state of mental health [17].

The COVID-19 pandemic has forced the transfer of face-to-face education to online education as a health measure while continuing teaching and learning processes. In this sense, teachers have had to adapt at an emergency rate to this new scenario, which involves the use of ICT to teach classes remotely. While this educational modality has pros where, the flexibility of schedules and spaces stands out, they also have cons, such as lack of social interaction with colleagues and teachers, lack of technological knowledge, quality of technological means and tools, and greater performance and dedication of time [18,19,20]. This is the reason that teacher’s role in the effective use of ICTs as an educational tool is relevant in crisis situations and will continue to be after COVID-19, when it is supposed to return to normal. To this end, it must have technical training and the technological means to assimilate the pedagogical uses of ICT that it employs [20].

In research prior to COVID-19, it was already shown that lack of adjustment between the teacher and the demands of technological environment was associated with the techno-stress experienced by teachers while using ICT into the classroom that, in line with Al-Fudail and Mellar [21], would arise from the lack of adequacy, which manifests itself with psychological, physical, and behavioral symptoms, between the demands of technological environment, such as preparing technology or correcting errors and the skills of teachers, and among the needs of teachers in terms of having adequate technology, training, and support, and the offer. In addition, they state in their study that teachers report coping strategies such as trying to correct mistakes and seek technical assistance and training and blaming themselves or managing their feelings to accept the situation. On the other hand, they mention that the inputs that did not satisfy the needs of teaching were found in the areas of technological performance and technical and social support, and the main causes of technostress found were the lack of adequacy when teachers were unable to deal with technological errors, increase in labor demands, and not being able to make an effective use of technology into the classroom due to lack of pedagogical preparation.

In accordance with Panisoara et al. [22], burnout and technostress have a negative effect on the intention of continuity of online instruction during COVID-19 pandemic, on the other hand, intrinsic motivation would have the opposite effect. In the absence of this, continuing teaching could be achieved only based on an extrinsic motivation such as the fear of losing work or the need for a salary that ensures basic needs. A teacher’s intrinsic motivation, in association with knowledge related to technology integration, can reduce an individual’s perception of difficulty in relation to online instruction. If teachers do not perceive themselves with self-efficacy in the use of technology, they could stop online instruction. Since intrinsic motivation in work would be positively associated with technological pedagogical knowledge and teacher self-efficacy, it is important to generate conditions that optimize skills acquisition in the use of technology, for which it is necessary to know the working conditions and technostress levels manifested by teachers at different educational levels.

Teachers’ perception of support for innovation by educational institutions increases motivation at work, positive emotions to use ICTs in their classes, satisfaction, and job engagement. In turn, motivation has a positive relationship with personal and work resources. It means that those with greater competences in the use of ICTs feel more motivated and, at the same time, perceive positive emotions regarding the use of ICTs, with greater satisfaction, and therefore, work commitment. So, fostering digital self-efficacy and institutional support are key to optimizing the use of ICTs [23].

The incorporation of ICTs into the educational field requires a curriculum reform that implies a change in work routines, since techno-stressors such as techno-insecurity, techno-invasion and techno-overload increase exhaustion in teachers. In contrast, facilitating digital literacy would mitigate the negative impact of exhaustion and stressors such as techno-complexity, techno-insecurity, techno-invasion, and techno-overload [24]. The ability to integrate technology into the curriculum and technical and social school support have positive effects on the reduction of technostress in high school teachers and influence the need to use technology [25].

Syvänen et al. [26] mention that high levels of technology in teachers are related to having lower skills, negative attitudes, and less frequency in the use of ICTs, and, on the other hand, when there is a lower concordance of ICT with the style of teaching and scarce school support in their use. They showed that more experienced teachers, women, and subject teachers, who teach older groups, compared to those in the classroom, were more stressed. The latter factor, related to demands and work resources, where there are different curricular requirements of the different teaching contexts, are associated with technostress. In addition, the most common sources of technostress among teachers were lack of education and lack of interest. They propose that ICT skills can be promoted so that more experienced teachers train to less experienced teachers or share knowledge.

In agreement with Wang and Li [27], university requirements related to the use of ICTs, their suitability, and maintaining the skills and needs of teachers are factors that affect job performance and organizational management that includes organizational demands in the use of ICTs and the resources available to teachers to meet these demands; they would determine technostress by exerting a negative influence on job performance, affecting teachers of higher grades more than those of lower grades. They mention that technostress would be determined by organizational aspects rather than technological; it is relevant that the choice of ICTs that must be introduced, the form of implementation, and availability of support according to the needs of teachers is made through participatory instances.

Li and Wang’s research [28] on the impact of techno-inhibitors and techno-creators on teachers’ job performance highlights that inhibitors such as facilitating participation and providing technical support have mitigation effects on techno-overload, techno-complexity, and techno-insecurity. At the same time, techno-complexity and techno-insecurity have a significant negative influence, and facilitating literacy and participation have positive effects on job performance. While these findings focus on university professors, similar outcomes are expected in primary and secondary school teachers, given the common scenario in which teachers have forcibly adapted to the use of ICT as the main tool for working and interacting with their students during the COVID-19 pandemic.

## 2. Materials and Methods

It is used as a measuring instrument for Technostress (RED-TIC) [10], which has been applied in Chile prior to the COVID-19 pandemic for professors [29] as a semantic calibration with an internal consistency (Cronbach’s alpha) of 94%. Additionally, following results [30] are observed in teachers: comparative fit index (CFI) = 0.900, root mean square error of approximation (RMSEA) = 0.103, Tucker–Lewis index (TLI) = 0.90, and standardized root mean square residual (SRMR) = 0.05 but based on a geographically reduced sample to only 2 of the 17 politic-administrative regions in which the country is divided and without the current intensity of the docent telework.

The data collection was carried out with sampling at the national level on a population of 308,556 teachers, according to data from the Ministry of Education in Chile as of December 2019 [31], together with the trade union of workers Chilean Teachers Association (“Colegio de Profesores y Profesoras de Chile”), achieving a *n* = 3006 responses with coverage of 289 communes of the 346 in which the country is administratively divided. Sample has been stratified according to the criteria of the Ministry of Science, Technology, Knowledge, and Innovation [32], in 6 macrozones (MZs), according to the territorial concentration of the population. Thus, 433 cases (14.4%) were surveyed in MZ-north, 445 cases in MZ-center (14.8%), 685 cases in MZ-south center (22.8%), 360 cases in MZ-south (12.0%), 32 cases, MZ-austral (1.1%), and 1051 cases in Metropolitan Region (35.0%).

To calculate the size, a confidence level of 95% was established, with a heterogeneity of 50% and a margin of error of 1.78, obtaining the sample of 3006 professors and professors. The random selection of cases was made from the records of the College of Professors and Professors within each MZ, from their communal representations. The survey was administered electronically (between September and November 2020), from the databases containing the teacher records as of August 2020 and the resulting data set is a Supplementary Material to this article.

To measure technostress, the instrument was first validated, using a Confirmatory Factor Analysis (CFA), which is used in previous studies of technostress [12,13,33,34,35,36]. In this case, the Lavaan package [37] of the RStudio statistical software (RStudio Team, Boston, MA, USA) has been used. Instrument constructs must meet the reliability of internal consistency, convergent, and discriminatory validity, and to identify model fit, recommended global measures are estimated; the criteria to be followed are set out in Table 1 and Table 2 [38,39,40,41,42,43,44].

The average variance extracted (AVE) considered in this case is one of the criteria used to perform the heterotrait-monotrait (HTMT) test [45]. This AVE describes the variance considering the items that make up the construct, to prove that the data set of these items contributes to the construct by explaining more than half of its variance (dispersion). Thus, since it is a CFA, the load of each item should only contribute to the construct in which it is included. On the other hand, divergent validity tests the relationship between constructs by comparing their correlations, with a value of (HTMT) less (<) than 0.90, it is possible to ensure that the constructs measure different concepts [41].

Then, to establish whether there are differences between technostress scales of each dimension and the gender of the teachers, a Chi-square test is performed, and finally, a proportion test is carried out to identify significant differences by gender on each scale [45].

## 3. Results

The sample of 3006 teachers with national coverage indicates that 27.4% of all education professionals are male, while 71.7% are for female teachers and 1% are classified as “other” gender. In addition, 58.30% are primary school teachers, 35.2% are from secondary education, and the remaining 6.5% are from adult education. On the other hand, only 3.9% of teachers work less than 22 h per week, while another 50.2% spend between 23 and 43 h. That is, 45.9% have indicated maintaining 44 h of work per week, which corresponds to a full working day, according to Chilean legislation.

Regarding working conditions, 67.1% have an indefinite contract, 31% maintain a fixed-term contract, and 1.9% of teachers indicated that they have a contract at fees for task performed or does not have a formal contract.

In terms of the working day, 52.1% of teachers teach only in the morning, 40.6% teach in mixed days (morning, afternoon, and evening), another 4.6% work exclusively in the afternoon, while 2.1% work only in evening, and 0.6% did not answer the question. Alongside the above, it is also interesting to note that surveyed teachers have on average 19.5 years of teaching experience, with an average age equal to 44.4 years and a median of 43 years. Finally, it is important to note that 90.2% of the teachers surveyed indicated working in a single establishment and 97.2% pointed they have a professional university degree.

About validity and reliability procedures, for this dataset, the model has a good absolute fit, since the SRMR value is 0.054 (<0.080), and the root mean square error of approximation (RMSEA) is 0.098. However, the p-value of the Chi-square test is 0, so the null hypothesis is rejected because there is no good adjustment between the variance matrix and sample covariance compared to the theoretical ones. However, this test is sensitive to sample size, so having 3006 observations overlaps the SRMR result [46]. Regarding comparative adjustment measures, the IFC and TLI both have a value of 0.99, so the model has a good fit.

**Figure 2:**
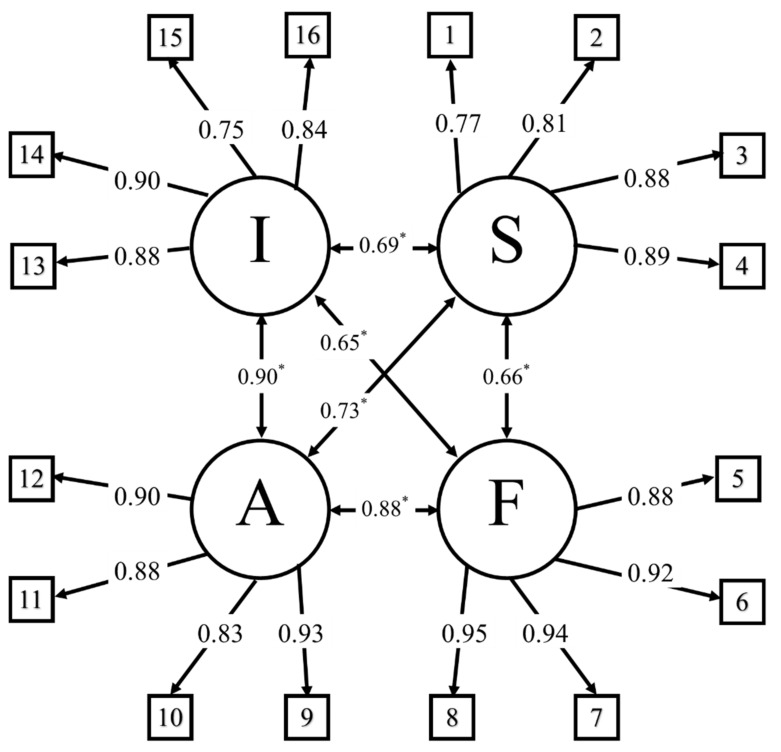
Confirmatory factor analysis for RED-TIC scale. * Double-headed arrows represent covariances in confirmatory factor analysis [41]. Factors are represented in circles, and variables 1 to 16 in boxes.

About the theoretical constructs or dimensions of technostress meet the criteria of internal consistency and convergent and discriminatory validation, as is shown in Table 3 and Figure 2, which details the structure of the factorial confirmatory analysis (CFA).

Figure 2 shows the results of the confirmatory factor analysis for the four factors that measure technostress on the RED-TIC scale [10,41]. Table 4 sets out the scores used to identify stress levels for each of the technostress dimensions and the result ratios [10].

### Moderating Variables

The study considered reviewing the possible differences between different attributes of teachers, i.e., reviewing variables such as education level at which teacher works, the weekly chronological hours of work in an educational establishment, type of employment contract, the day of the day when he mostly teaches classes, years of work in education, number of educational establishments in which he works, possession of professional title, and age. However, statistical procedures did not show for this set of variables statistically significant differences, which allow to distinguish differences in the manifestations of technostress.

As an example, see Table 5, the analysis of the day’s shift variable in which teachers teach, the results of the Kruskal–Wallis test give a *p*-value < 0.05 only for the fatigue dimension, so the null hypothesis is rejected that there are no differences between the day’s shift. See Table 5, there are differences in the very low and medium (low) stress levels for the teachers who work in both daytime and those who work in the evening.

In this way, statistically significant differences were found only in the very low and medium low levels of the fatigue dimension. This would be showing that percentage of teachers who work on an evening workday present a higher level of fatigue compared to teachers working on the morning along with evening. That is, while differences were found, these are very marginal to point out that one group is more techno stressed than the others.

As for the gender variable, the analysis showed a more interesting scenario, which, in turn, contributes as a moderator to possible data variability problems due to the low response of male teachers within the surveyed population. Due to this, it was decided to carry out the Kruskal–Wallis test of differences in proportions, for each dimension(factor) of the RED-TIC scale. This test identifyies possible differences within the sample, and allows to control for differences in the size of each subsample. In this way, the results of the Chi-square test deliver a *p*-value < 0.05, in each case, so the null hypothesis is rejected that there are no differences between the gender and the levels of each techno-stress dimension (see Table 6).

Thus, giving gender openness to Table 6, in anxiety dimension at the level of high anxiety, it is identified that the proportion of women is significantly higher than that of men, and, at the level of very low anxiety, the proportion of men is higher than that of women. Significant differences are found in medium-low levels and very high skepticism, the proportion of women with medium low skepticism level is higher than men (0.202 vs. 0.258). Differences in very low and very high inefficiency levels are significant, so the proportion of men is higher than for women at these levels. Finally, there are significant differences in levels of very low, high, and very high fatigue; at the level of very low fatigue, the proportion of men is higher than women, and at high and very high fatigue levels, the proportion of women is higher than men (see Table 7). The proportions for high (H) and very high (VH) levels that indicate the levels of concern for the different dimensions (factors), manifestations (combination of factors), and technostress in general are calculated. The results shows that techno anxiety exist in 11.0% of all teachers, in which in male teachers the rate reaches 11.5% and in female teachers stands at 10.8%. Regarding techno fatigue, the results point to a 7.2% of total affected teachers, in which 6.5% and 7.4% of male and female teachers shows this manifestation, respectively. Accordingly, the total percentages of teachers who are affected by at least one of the 2 manifestations is 11.6%, and worryingly, 6.8% are jointly affected by both manifestations, which is 7.0% of female teachers and 6.4% of male teachers.

## 4. Discussion

In terms of validation procedures and results and scale adjustments, both the instruments applied and values and scores match with others recent research. In this way, in general terms, the other technostress measurement scales and the values of this research are like the studies of the last decade [30,33,34,35]. The previous local study focused on technostress of teachers in a prepandemic context [30]. However, in the three referenced research cases, the sample sizes are narrower (441, 267, and 537 cases reviewed) and, at the same time, they do not correspond to a study with a sample at the national level. Objectives of other technostress studies, published in 2020, with relative samples including 1462 cases [12] and 1744 cases [13], focused on the techno stressors of higher education students.

However, as a limitation, the local case studied is a recent research, and that is why, it has been chosen to obtain concrete measures and a multivariate analysis that focuses on confirming the empirical validity of the RED-TIC scale before being able to conclude the relation to underlying variables. At this point, we distanced ourselves with other recent studies that seek to test theory through an analysis with partial least square structural equation modelling (PLS-SEM) [47] in K-12 teachers [24] and in lecturers and professors [28]. However, this study is in line with articles that use confirmatory factorial analysis (CFA) to analyze technostress of university students [9,10]. On the other hand, while technostress studies in teachers are scarcer, it is interesting to note that the results of the constructs and procedures validation used are also consistent with recently published research on teachers [22,36].

Regarding differences by technostress dimensions and manifestations, in the context of the COVID-19 pandemic and under a teleworking or teleteaching scheme, the results showed that female teachers suffer higher fatigue and anxiety with the use of technology as an educational means. Such manifestations of technostress, because of these emerging psychosocial risks present at work, associated with the use of ICTs, are also observed in Salanova’s research [10]. On the other hand, it is important to emphasize that, according to the latest psychosocial risk assessment, through the application of the SUSESO/ISTAS 21 questionnaire, in work centers in Chile during 2019, the results of which were published in 2020, in a scenario prior to COVID-19 pandemic, most of the psychosocial risk variables considered show differences according to gender. Thus, women in this country are more exposed to these risks with significant differences in relation to men in 13 out of 19 subdimensions, which correspond to emotional psychological demands, influence, development possibilities, sense of work, clarity of role, role-playing conflict, leadership quality, relationship with superiors, relationship with peers, esteem, contract insecurity, job insecurity, and double presence [48]. This reinforces the idea that female gender would have reason to be more stressed, consistent with this study where the level of technostress, simultaneously techno anxiety and techno fatigue, is 7%, greater than that of men reaching 6.4%. The need to reconcile domestic and professional tasks due to the establishment of telework and the closure of schools, with assistance in educational tasks for children could be related to these results. According to the study by Lambert et al. [49], work–family conflict influences levels of work stress in women. Additionally, La Torre et al. [50] state that women experience greater techno-overload, techno-invasion, techno-complexity, and role overload than men.

On the other hand, the proportion of techno anxiety (11.0%), which surpasses Techno fatigue (7.2%), tends to be coincident with the findings of Estrada et al. [30] prior to COVID-19 pandemic and teleworking resulting from the quarantine of confinement, but the results of both manifestations have a higher distance, and the total level of techno-stresses is reduced in this sample greater than 6.8%. García-Gonzalez’s study [51] also shows coincidences, although this research focuses on instrument validation, more than in technostress measuring and moderating variables comparison. It is important to consider that the vast majority of the teachers who answered the survey have formal education, which, according to Tarafdar et al. [52], would be related to lower technostress levels.

In relation to future lines of research and deepening, it is relevant to design educational public policies, including other levels of school and members of educational communities, such as students, administrators, and other education-assistant professionals. In the same manner, it is important to delve into the effects of techno-inhibitors and techno-creators on technostress in education in an empirical way [28,36] and, in reality, compared to other countries. Similarly, it is necessary to delve into the moderating variables and their effects on techno-inhibitors and techno-creators [52,53], and to explore what are the main conditions that predispose female gender people to present greater manifestations of technostress, in the teleteaching context, compared to those of the male gender [54].

## 5. Conclusions

This research, when measuring the levels of technostress in a national sample of teachers of different levels of primary and secondary education, has allowed to account for differences in dimensions that show manifestation of technostress. At the same time, as the results warn, statistically significant differences have been appreciated when the gender variable is introduced. In this way, in the case of Chilean primary and secondary school teachers, it is advised that female teachers show a higher technostress than their male gender pairs, a condition that is also consistent with other contemporary studies that have analyzed these differences.

In this way, the results of this research acquire a relevant importance to strengthen knowledge on the subject, especially when in the case of teachers, keys are collected for the formulation of strategies and measures that allow an effective and sustainable integration of ICTs in the educational field, an integration that undoubtedly transcends the juncture that has installed COVID-19 pandemic.

For contemporary educational systems, the integration of information and communication technologies (ICTs) into education has become an urgent necessity both for universal access to knowledge and for pedagogical and didactic resources associated with information and communications technologies. This implies a transformation in how teaching work is organized and how teachers’ skills are developed and consolidated when using and incorporating ICTs, without the increasing of stress associated with their use. In this sense, the findings of this research are a concrete contribution to the design of educational policies, generating the beginning of a way to study other factors, in addition to those related to the use of ICT, to generate conditions that optimize work [55].

Specifically, this research infers the demands and resources of work with technological means in the educational field, where teachers from primary and secondary schools develop daily warning of the risks of stress and pathological exhaustion. This confirmed that work in primary and secondary schools, especially in urban areas, relates to greater manifestations of stress, especially in the case of female teachers.

In this regard, the establishment of public policies focused on mitigating those stressful factors, beyond human-ICT interaction, is recommended. According to Ayyagari et al. [56], these factors are associated with tasks (work overload, work schedule, and exposure to risks and dangers), role characteristics (ambiguity, conflict, and overload), interactions within the organization (interpersonal relationships and style of leadership), career (job insecurity and career advancement), organizational factors (climate and structure), work–home interface (work–home conflict and invasion of privacy), and characteristics related to the physical work environment. To which, Tarafdar et al. [57] add that the attitude towards ICT, the workload, the complexity of the work, digital literacy, and user participation affect the perception of technostress.

Finally, in practical terms, the results of this research contribute to analyzing the effective implementation of the Chilean Educational Reform, in particular, Law 20.903 [58], which creates the System of Professional Teacher Development and sets the criteria to guide the training of teachers and the conditions of professional practice. Additionally, in article 1 of this law, it is stated as a purpose to contribute to the continuous improvement of the teaching professional performance and the systematic reflection of professional practice. Thus, contributing to an explicit demand of the Chilean Teachers Association, to identify manifestations of risk in the mental health of teachers and based on these findings, this research propose actions and strategies that attend and prevent emotional or mental illnesses. Additionally, in theoretical terms, this research gather evidence that allows the development and promotion of didactic and pedagogical adjustments that, while safeguarding the working conditions of the teaching activity, allow the consolidation of an educational model that combines the advantages of face-to-face teaching with classes supported by virtual resources, both synchronous and asynchronous.

## Figures and Tables

**Table 1:** Validation measures of the theoretical constructs.

**Convergent Validity**	Load	>0.70
	Communality	>0.50
	Average variance extracted (AVE)	>0.50
**Discriminant (Divergent) Validity**	Heterotrait-monotrait ratio of correlations (HTMT)	<0.90
**Internal Consistency Reliability**	Cronbach’s alpha	0.70–0.90
	Composite reliability (CR)	0.70–0.90

**Table 2:** Global measures of scale validation.

Adjustment Indices		Quality of Model Adjustment		
Chi-squared test	*p*-value	>0.05 Good		
Standardized root mean square residual	SRMR	<0.08 Good		
Root mean square error of approximation	RMSEA	≤0.05 Very good	0.05 < RMSEA ≤ 0.08 Good	0.08 < RMSEA ≤ 0.10 Suffering
Comparative fit index	CFI	≥0.95 Very good	0.90 ≤ CFI < 0.95 Good	0.80 ≤ CFI < 0.90 Suffering
Tucker–Lewis index	TLI	≥0.95 Very good	0.90 ≤ CFI < 0.95 Good	0.80 ≤ CFI < 0.90 Suffering

**Table 3:** Global measures of scale validation.

Factor	Variable *	Convergent Validity			Discriminant Validity	Internal Consistency Reliability	
		Load	Communality	AVE	HTMT	Cronbach Alpha	CR
Skepticism (S)	r_1	0.767	0.589	0.704	0.667	0.856	0.905
	r_2	0.812	0.659				
	r_3	0.879	0.773				
	r_4	0.893	0.797				
Fatigue (F)	r_5	0.880	0.774	0.853	0.868	0.942	0.959
	r_6	0.923	0.853				
	r_7	0.936	0.875				
	r_8	0.953	0.909				
Anxiety (A)	r_9	0.932	0.868	0.785	0.804	0.899	0.931
	r_10	0.834	0.695				
	r_11	0.877	0.768				
	r_12	0.899	0.809				
Inefficacy (I)	r_13	0.883	0.78	0.713	0.722	0.861	0.911
	r_14	0.902	0.813				
	r_15	0.749	0.561				
	r_16	0.835	0.697				

* See details in Appendix A.

**Table 4:** Scores correction by levels for the sample of teachers and teachers’ ratio (TR) by technostress dimension (*n* = 3006).

Levels ^a^	%	Skepticism	TRS *	Fatigue	TRF *	Anxiety	TRA *	Inefficacy	TRI *
Very low	>5%	0.00	0.000	0.00	0.081	0.00	0.146	0.00	0.243
Low	5–25%	0.00	0.262	0.01–1.00	0.170	0.00–0.50	0.114	0.01–1.00	0.278
Medium (low)	25–50%	0.01–1.00	0.243	1.01–3.00	0.252	0.51–1.75	0.257	1.01–1.50	0.108
Medium (high)	50–75%	1.01–2.75	0.264	3.01–5.00	0.295	1.76–3.75	0.247	1.51–2.50	0.149
High	75–95%	2.76–5.00	0.189	5.01–5.99	0.108	3.76–5.75	0.194	2.51–4.74	0.178
Very high	>95%	>5	0.043	>5.99	0.094	>5.75	0.042	>4.75	0.045
Mean		1.61	–	3.07	–	2.21	–	2.87	–
Standard Deviation		1.65	–	2.00	–	1.87	–	1.57	–

a. Levels according to the scaling and normalization of scores obtained with the RED-TIC scale [10]; *. Teachers’ ratio in percentage of teachers classified at each level for each dimension of the RED-TIC scale (factor).

**Table 5:** Teachers’ ratio by technostress dimension Fatigue (*n* = 3006).

Levels	Both Daytime Journeys	Evening Journey
Very low	0.074 *	0.156 *
Low	0.167	0.172
Medium (low)	0.235 *	0.359 *
Medium (high)	0.294	0.188
High	0.115	0.031
Very high	0.115	0.094

* Statistically significant differences.

**Table 6:** Chi-square test *p*-value for differences in gender proportions by dimension (factor) of the RED-TIC scale.

Dimension	*p*-Value
Skepticism	0.040 *
Fatigue	0.003 *
Anxiety	0.024 *
Inefficacy	0.000 *
Techno anxiety	0.491
Techno fatigue	0.505

* Statistically significant differences.

**Table 7:** Test of proportions for level by gender in technostress, manifestations and dimensions.

Levels	Skepticism		Fatigue		Anxiety		Inefficacy		Techno Anxiety		Techno Fatigue		Techno Stress	
	M	F	M	F	M	F	M	F	M	F	M	F	M	F
Very low	0.000	0.000	0.105 *	0.073 *	0.181 *	0.136 *	0.272 *	0.234 *	–	–	–	–	–	–
Low	0.278	0.260	0.167	0.174	0.119	0.118	0.262	0.286	–	–	–	–	–	–
Medium (low)	0.202 *	0.258 *	0.293	0.241	0.253	0.259	0.117	0.105	–	–	–	–	–	–
Medium (high)	0.285	0.258	0.293	0–294	0.253	0.243	0.133	0.154	–	–	–	–	–	–
High (H)	0.189	0.188	0.084 *	0.115 *	0.170 *	0.205 *	0.171	0.182	–	–	–	–	–	–
Very high (VH)	0.058 *	0.037 *	0.071 *	0.103 *	0.036	0.044	0.058 *	0.040*	–	–	–	–	–	–
H + VH	0.247	0.225	0.155 *	0.218 *	0.205 *	0.248 *	0.229	0.221	0.115	0.108	0.065	0.074	0.064	0.070
Dominant gender	–	–	–	F(+)	–	F(+)	–	–	–	–	–	–	–	–

* Statistically significant differences; M: male, F: female.

## Data Availability

The analyzed dataset has been anonymized and included as Supplementary Materials.

## Supplementary Materials

The following are available online at https://www.mdpi.com/article/10.3390/ijerph18105458/s1, Table S1: TSPTW.xlsx.

## Appendix A

Technostress theoretical constructs by NTP730. This appendix presents the RED-TIC scale, originally published in Salanova et al. [59] as a Technical Note on Prevention (NTP730) and adapted for the first time for its use to the context of Chile by Vega et al. [29].

**Factor: Theoretical Constructs**	**Variable**	**Components**
Skepticism	r_1	S1. With the time passage, technologies interest me less and less
	r_2	S2. Every time I feel less involved in the use of ICT
	r_3	S3. I am more skeptical about the technology’s contribution in my work
	r_4	S4. I doubt the working meaning with these technologies
Fatigue	r_5	F1. I find it difficult to relax after a workday using them
	r_6	F.2 When I finish working with ICT, I feel exhausted
	r_7	F3. I’m so tired when I just work with them that I cannot do anything else
	r_8	A3. I doubt when using technologies for fear of making mistakes
Anxiety	r_9	A1. I feel tense and anxious when working with technologies
	r_10	A2. It scares me to think that I can destroy a lot of information by the improper use
	r_11	A3. I doubt when using technologies for fear of making mistakes
	r_12	A4. Working with them makes me feel uncomfortable, irritable, and impatient
Inefficacy	r_13	I1. In my opinion, I am inefficient using technologies
	r_14	I2. It is difficult to work with information and communication technologies
	r_15	I3. People say that I am inefficient using technologies
	r_16	I4. I am unsure of finishing my tasks well when I use ICT
